# Up-regulation of *Arl4a* gene expression by broccoli aqueous extract is associated with improved spermatogenesis in mouse testes

**DOI:** 10.7705/biomedica.5962

**Published:** 2021-12-15

**Authors:** Omid Jazayeri, Setareh Farahmand Araghi, Tahereh A. Aghajanzadeh, Fereshteh Mir Mohammadrezaei

**Affiliations:** 1 Department of Molecular and Cell Biology, Faculty of Basic Science, University of Mazandaran, Babolsar, Iran University of Mazandaran Department of Molecular and Cell Biology Faculty of Basic Science University of Mazandaran Babolsar Iran; 2 Department of Plant Sciences, Faculty of Basic Science, University of Mazandaran, Babolsar, Iran University of Mazandaran Department of Plant Sciences Faculty of Basic Science University of Mazandaran Babolsar Iran; 3 Department of Animal Sciences, Faculty of Basic Science, University of Mazandaran, Babolsar, Iran University of Mazandaran Department of Animal Sciences Faculty of Basic Science University of Mazandaran Babolsar Iran

**Keywords:** Brassica, spermatogenesis, gene expression, mice, cadmium, Brassica, espermatogénesis, expression génica, ratones, cadmio

## Abstract

**Introduction:**

Broccoli (*Brassica oleracea*) is well known for its properties as an anticancer, antioxidant, and scavenger of free radicals. However, its benefits in enhancing spermatogenesis have not been well established.

**Objective:**

To study broccoli aqueous extract effects on sperm factors and the expression of genes *Catsper1*, *Catsper2*, *Arl4a*, *Sox5,* and *Sox9* in sperm factors in mice.

**Material and methods:**

Male mice were divided randomly into six groups: (1) Control; (2) cadmium (3 mg/kg of mouse body weight); (3) orally treated with 200 µl broccoli aqueous extract (1 g ml^-1^); (4) orally treated with 400 µl of broccoli aqueous extract; (5) orally treated with 200 broccoli aqueous extract plus cadmium, and (6) orally treated with 400 µl of broccoli aqueous extract plus cadmium. We analyzed the sperms factors and *Catsper1*, *Catsper2*, *Arl4a*, *Sox5,* and *Sox9* gene expression.

**Results:**

An obvious improvement in sperm count and a slight enhancement in sperm motility were observed in mice treated with broccoli extract alone or with cadmium. Sperm viability was reduced by broccoli extract except for the 200 µl dose with cadmium, which significantly increased it. Interestingly, *Arl4a* gene expression increased in the 400 µl broccoli- treated group. Likewise, the *Arl4a* mRNA level in mice treated with cadmium and 200 µl of broccoli extract was higher than in the cadmium-treated mice. Furthermore, broccoli extract enhanced the mRNA level of *Catsper2* and *Sox5* genes in mice treated with 200 µl and 400 µl broccoli extract plus cadmium compared with the group treated solely with cadmium.

**Conclusion:**

The higher sperm count in broccoli-treated mice opens the way for the development of pharmaceutical products for infertile men.

## Resumen

**Introducción.:**

El brócoli (*Brassica oleracea*) se conoce por sus propiedades como anticancerígeno, antioxidante y eliminador de radicales libres. Sin embargo, sus beneficios en la espermatogénesis aún no se han determinado suficientemente.

**Objetivo.:**

Estudiar los efectos del extracto acuoso de brócoli sobre los factores espermáticos y la expresión de los genes *Catsper1*, *Catsper2*, *Arl4a*, *Sox5* y *Sox9* en ratones.

**Materiales y métodos.:**

Los ratones machos se dividieron aleatoriamente en seis grupos: 1) control; 2) tratados con cadmio, 3 mg/kg de peso corporal; 3) tratados con 200 µl de extracto acuoso de brócoli (1 g ml^-1^); 4) tratados con 400 µl de extracto acuoso de brócoli; 5) tratados con 200 µl de extracto acuoso de brócoli más cadmio, y 6) tratados con 400 µl de extracto acuoso de brócoli más cadmio. El extracto acuoso de brócoli se administró por vía oral. Se analizaron los factores espermáticos y la expresión de los genes *Catsper1*, *Catsper2*, *Arl4a*, *Sox5* y *Sox9.*

**Resultados.:**

Se observó una mejoría obvia en el recuento y una ligera mejoría en la motilidad de los espermatozoides, en ratones tratados con extracto de brócoli solo o con cadmio. La viabilidad de los espermatozoides se redujo con el extracto de brócoli, excepto con la dosis de 200 µl más cadmio, la cual la aumentó significativamente. Curiosamente, la expresión del gen *Arl4a* aumentó en el grupo tratado con 400 µl del extracto. Asimismo, el ARNm del *Arl4a* en ratones tratados con cadmio y 200 µl del extracto, fue más abundante que en los ratones tratados solo con cadmio. Además, el extracto de brócoli aumentó la cantidad de ARNm de los genes *Catsper2* y *Sox5* en ratones tratados con 200 y 400 µl de extracto de brócoli más cadmio, en comparación con el grupo tratado únicamente con cadmio.

**Conclusión.:**

El mayor número de espermatozoides en ratones tratados con brócoli abre el camino al desarrollo de productos farmacéuticos para hombres infértiles.

Infertility is defined by the failure to achieve a clinical pregnancy after one year or more of regular unprotected sexual intercourse. Approximately 40-50% of all infertility cases are due to male factors ^1^. While reproductive technologies can counter male infertility, genetic defects may still be passed to the male’s offspring ^2^.

Medicinal plants are used for the development of new drugs, as well as for health care. For example, *Trigonellae* Semen, derived from *Trigonella foenum-graecum* L., is commonly used as a medicinal herb for the treatment of infertility in Korean medicine ^2^. *Panax ginseng* is a traditional medicinal plant for male infertility and rats treated with ginseng had had a significant increase in sperm count and sperm motility ^3^^,^^4^. *Aspalathus linearis* and *Camellia sinensis* have also a forceful positive impact on sperm factors ^5^. Decursin, extracted from *Angelica gigas*, has also shown a positive effect on sperm counts and motility in cryptorchidism-induced infertile rats ^6^. The protective effect of *Crocus sativus* L. on cadmium (Cd) toxicity in rat spermatogenesis has been reported as well ^7^. Besides, recent evidence suggests that the Ca^2+^ levels of mice sperm cells treated with *P. ginseng* extract increased significantly compared with the normal group inducing the expression of CatSper genes (involved in sperm motility and fertility) ^8^^,^^9^.

There is a growing body of literature on the therapeutic effects of cruciferous plants like broccoli largely attributed to their high content of glucosinolates. Broccoli is well known worldwide for its anti-cancer effects ^10^ and its antioxidant properties, which make it the finest natural active substance for scavenging free radicals ^11^^,^^12^, as an antigenotoxic agent ^13^, a reducer of fasting blood glucose in type 2 diabetes patients ^14^, a protection agent of the cardiovascular ^15^ and central nervous systems, against diabetic nephropathy and neuropathy ^16^, beneficial in the restoration of skin integrity ^17^, against *Helicobacter pylori* infection ^18^, and for the improvement of social interaction in patients with autism ^19^.

Heavy metal pollutants such as cadmium and lead are considered sources of significant environmental damage as they do not have any biological functions and can be extremely toxic even at low concentrations ^20^. The toxic effect of heavy metals on living organisms’ health are expressed in diseases and conditions like cancer, infertility, nephritis, hair loss, brain damage, cardiovascular disease, low blood pressure, and paralysis. However, kidney and lung damage, fragile bones, and calcium deregulation in biological systems have been determined as specific toxicological effects of cadmium ^21^.

The effect of broccoli on spermatogenesis and related gene expression in mouse testes has yet to be determined. The genetic bases and molecular mechanisms underlying spermatogenesis and its molecular regulation are not fully understood yet. In this context, in the present study, we analyzed the impact of broccoli extract on sperm count and the expression of *Arl4a* gene (involved in spermatogenesis) ^22^, of *Catsper1* and *Catsper2* genes (involved in sperm motility) ^23^, as well as that of *Sox5* and *Sox9* genes (transcription factors involved in the Sertoli cells and sex development) ^24^.

## Materials and methods

### Experimental animals

The study was approved by the Ethics Committee at the University of Mazandaran, Iran (IR.UMZ.REC.1399.002). We purchased NMRI male mice (5-6 weeks old between 25-30 g of weight) from the Pasteur Institute (Tehran, Iran), which were kept for one week in polycarbonate cages for adaptation to their new environment until reaching the desired conditions. They were kept under standard conditions (12 h light/dark cycle at 24 ± 2°C) with free access to drinking water and standard pellets. Once every two days, the cages were cleaned, the remaining food was collected, and fresh food was provided. Finally, they were anesthetized intraperitoneally with ketamine hydrochloride (100 mg kg^−1^) and xylazine (5 mg kg^−1^).

### Preparation of the broccoli aqueous extract

Two-month broccoli was collected from a local greenhouse, immediately frozen and transferred to the plant physiology laboratory. Broccoli flowers were grinned in liquid nitrogen to a fine powder, dissolved in boiling water, and boiled for 3 minutes (1 g ml^-1^). Then, the suspension was filtered through one layer of filter paper and the extraction was centrifuged at 10,000*g* for 10 minutes at 4°C and stored at −20°C for further experiments ^25^.

### Experimental design

Thirty-six NMRI male mice were divided randomly into the following six groups, with six animals per group: 1) control; 2) cadmium, 3 mg/kg of mouse body weight; 3) orally treated mice with 200 broccoli aqueous extract (1 g ml^-1^); 4) orally treated with 400 µl of broccoli aqueous extract; 5) orally treated with 200 broccoli aqueous extract plus cadmium, and 6) orally treated with 400 µl of broccoli aqueous extract plus cadmium. The mice in the control group only received distilled water. In the cadmium group, mice were injected intraperitoneally only with cadmium chloride (3 mg/kg of mouse body weight). Four of the groups were orally administered 200 and 400 µl of the extract (1 g ml^-1^/60 g of mouse body weight) with and without intraperitoneally injection of cadmium chloride. The extract was administered every day for 48 days and cadmium on the last day of the treatment. On the 49th day of the experiment, all animals were sacrificed and samples were collected. The sperm was rapidly detached from the epididymis and used to determine the parameters. Mice testis were separated, immediately frozen in liquid nitrogen, and stored at -80°C for subsequent RNA extraction.

### Evaluation of cauda epididymal sperm count and motility

We used the seminal plasma aspirated from the caudal part of the mice epididymis for the semen analysis. The epididymis was put in a solution containing 1 ml of PBS buffer (pH=7) and then cut into small pieces. We incubated the tissue homogenate at 37°C for 10 minutes to release the sperm in the solution. We placed 10 μl of the sperm on a clean slide coverslip for sperm count and motility evaluation ^26^. Total sperm counts were determined with a hemocytometer under a light microscope at 200X magnification. Spermatozoa were collected with a micropipette and placed on a slide for motility estimation and classification into motile and non-motile sperms. Motility was expressed as the percentage of sperm showing movement (fast forward, slow movements, and movement in place); data were expressed as percentages ^26^.

### Sperm viability

Viability was assessed by eosin-nigrosin solution ^26^: A 10 µl sample of the sperm suspension was placed on a glass slide, mixed with 10 µl eosin, and after drying at laboratory temperature observed under a light microscope (400X). With this procedure, the head of the dead spermatozoa absorbs eosin and becomes red, but live spermatozoa appear colorless as the plasma membrane remains intact. Live spermatozoa were counted in five fields of vision at random and their percentage was recorded.

### Semi-quantitative RT-PCR

*RNA extraction and cDNA synthesis.* Total RNA was extracted from the mice testis using RNX plus (EX6101) solution following the manufacturer’s protocol (SINACLON, Iran). The RNA pellet was dissolved in RNase-free water (DEPC treated water). Total RNA was then treated with DNAaseI (SINACLON, Iran) to destroy possible genomic DNA contamination followed by heat treatment (65°C for 10 minutes) to inactivate the enzyme and then stored at −80°C for future cDNA synthesis. We checked the purity and integrity of the total RNA extracted at 260/280 nm ratio using a Thermo Scientific NanoDrop spectrophotometer and visualized it on 1% agarose gel. We used a commercial cDNA synthesis kit (2-steps RT-PCR kit, Vivantis, Malaysia) following the manufacturer’s instructions. Briefly, 1 µg of total RNA, 0.5 µl M-MuLV Reverse Transcriptase (200 U/µl), 2 µl of 10X Buffer M-MuLV, 1 µl Oligo d(T)18 (40 µM), 1 µl of 10 mM dNTPs were added to a 0.2 ml microcentrifuge tube. The reaction mixture was adjusted to the final 20 µl volume with nuclease-free water.

### Polymerase Chain Reaction (PCR)

Primer sequences, GeneBank ID, and amplification product sizes are summarized in table 1. Primers were synthesized (Metabion, Germany) and samples were normalized with the β *actin* gene as a reference gene for its constitutive expression. Synthesized cDNA was amplified by PCR reaction performed by using 2x PCRBIO Taq Mix Red in a reaction volume of 12.5 μl containing 6.25 μl 2x PCRBIO Taq Mix Red, 0.5 μl of each of the forward and reverse primers (10 pM), 1.5 μl cDNA, and 3.75 μl H_2_O. PCR conditions for *Catsper1*, *Catsper2* and *Sox9* were: 94°C for 2 minutes followed by 36 cycles (94°C for 30 s, 57°C for 30 s, and 72°C for 1 min as an extension), and a final extension at 72°C for 10 min. For *Sox5*, the conditions were: 94°C for 2 minutes followed by 32 cycles (94°C for 30 s, 60°C for 30 s, and 72°C for 1 minute as an extension), and a final extension at 72°C for 10 minutes. For *Arl4a* and β *actin*, the conditions were: 94°C for 2 minutes followed by 30 cycles (94°C for 30 s, 57°C for 30 s, and 72°C for 1 minute as an extension) and a final extension at 72°C for 10 minutes.

**Table 1:** Sequence of the designed primers used for RT-PCR

Gene	Function	Accession number		Primer sequences	Product size	Optimized cycle number*	Reference
*CatSperl*	Sperm motility	NM_	139301.3	Forward: 5' 'TCGGAGAACCACAGAGAAGAG-3'	566 bp	36	^27^
				Reverse: 5' CACACACCGGGAATATCTTC-3'			
*CatSper2*	Sperm motility	NM_	153075.3	Forward: 5' TGGCCACAGAGCAGTATTTG-3'	513 bp	36	^27^
				Reverse: 5' TGTCAGGCTGTTGCTTTGTC-3'			
*Arl4a*	Spermatogenesis	NM	007487.3	Forward: 5' CAGGCTGCAGTTCAACGAAT-3'	377 bp	30	Current study
				Reverse: 5' - AATGCCAAGGAGTCGATGAG-3'			
*Sox5*	Transcription factor	NM	001113559.2	Forward: 5' CCCCACATAAAGCGTCCAATG-3'	196 bp	32	^28^
				Reverse: 5' - TCTCCAGGTGCTGTTTGCTGAG-3'			
*Sox9*	Transcription factor	NM	011448.4	Forward: 5' GAAGCTGGCAGACCAGTACC-3'	479 bp	36	^29^
				Reverse: 5' - CTGCTCAGTTCACCGATGTC-3'			
*β actin*	Reference gene	NM	007393.1	Forward: 5' 'GGGAAATCGTGCGTGACAT - 3'	385 bp	30	^27^
				Reverse: 5' TCAGGAGGAGCAATGATCTTG -3'			

The number of PCR cycles was determined to obtain a detectable signal without reaching saturation.

We determined the number of PCR cycles for each gene to obtain a detectable signal without reaching saturation. For the electrophoresis of amplified products, we used 1% agarose gel and the amplified cDNA fragment was visualized and photographed under UV light. The band intensities were semi-quantitatively analyzed using the E-capt software (Vilber Lourmat, France) and normalized against that of β *actin*. The resulting data were expressed as means and standard deviation (SD) of at least three PCR replicates.

### GeneMANIA in silico analysis to study protein-protein interaction

We utilized a large set of protein-protein interaction databases within the GeneMANIA package ^30^ to build a protein-protein interaction network. This package comprises 244 databases/articles that collectively utilize experimentally proved physical protein-protein interactions in humans. The *Arl4a* gene was used as input for the GeneMANIA algorithm. Although we used “mice” in the study, we selected “*Homo sapiens*” as the organism in the GeneMANIA input setting because protein-protein interaction in “human” is more complete than in “mouse” and, thus, it provided a more comprehensive insight into *Arl4a* biological functions.

### Statistical analysis

All data were analyzed using the Prism software, one-way ANOVA, and Tukey post-test at a significance level of p≤0.05.

## Results

### Impact of the broccoli extract on the body and testis weight with and without Cd-toxicity

No significant difference was evident between groups regarding body and testis weight (table 2).

**Table 2:** Body and testis weight in NMRI male mice treated with 200 and 400 µl of broccoli extract (BE) of (1 g ml^-1^) with and without Cd (1 mg kg^-1^ of mouse weight). Data represent the mean of six mice in each group (± SD). The different letters indicate a significant difference among groups (p ≤ 0.05; Oneway ANOVA; Tukey´s HSD all-pairwise comparisons as a post-hoc test).

Sample	Control	Cd	BE (200 μl)	Cd+ BE (200 μl)	BE (400 μl)	Cd+ BE (400 μl)
Body weight (g)	47.7±3.9a	39.7±5.2a	38.7±4.8a	44.8±5.7a	40.9±1.5a	45.2±5a
Testis weight (g)	0.17±0.03a	0.16±0.04a	0.13±0.01a	0.15±0.03a	0.14±0.02a	0.16±0.02a

### Impact of the broccoli extract on sperm parameters with and without Cd- toxicity

**Figure 1:**
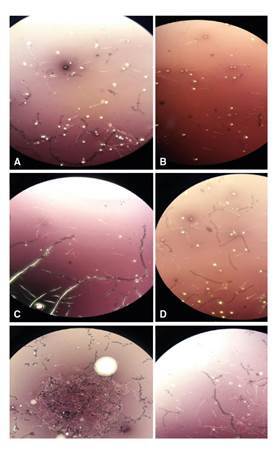
Microscopic images of sperm in NMRI mice treated with Cd and BE. A: Control; B: Cd; C: 200 µl of BE (1 g ml-1); D: 200 µl of BE (1 g ml-1) plus Cd (3 mg kg−1 of mouse body weight); E: 400 µl of BE (1 g ml-1); F: 400 µl of BE (3 g ml-1) plus Cd (1 mg kg−1). 4X

**Figure 2:**
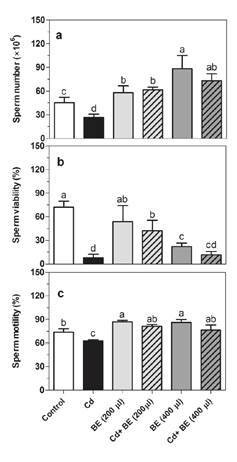
Sperm count (a), sperm viability (b), and sperm motility (c) in NMRI male mice treated with 200 and 400 µl of BE (1 g ml-1) with and without Cd (1 mg kg−1 of mouse weight). Data represent the mean of six mice in each group (± SD). Different letters indicate significant differences among groups (p ≤ 0.05; Oneway ANOVA; Tukey´s HSD all-pairwise comparisons as a post-hoc test).

Figure 1 displays sperm microscopic images in NMRI mice treated with cadmium and the broccoli extract while figure 2 shows the experimental data on sperm parameters: we observed a positive significant impact of the extract on the sperm count in a dose-dependent manner (figure 2A). The sperm count in the 400 µl extract-treated group was 1.5, i.e., two-fold higher than in the 200 µl extract-treated and the control groups (figure 1, A, C and E). Additionally, we found a clear benefit of the extract in the prevention of Cd-toxicity in the sperm count. Likewise, the sperm count significantly increased in the extract (almost 2.5 fold) and CD-treated (2.7 fold) groups (200 and 400 µl) compared to the mice treated only with Cd (figure 1B, D, F).

Furthermore, sperm viability was clearly reduced in Cd-treated mice compared with the control group (figure 1A, B; figure 2B). When Cd-treated mice were administered 200 and 400 µl (1g/ml) broccoli extract, the sperm viability increased 5.3 and 1.5 fold, respectively (figure 2B).

However, sperm viability hardly changed in mice treated with 200 µl extract and acutely decreased in mice treated with 400 µl extract (figure 2B). Additionally, sperm motility significantly decreased in Cd-treated mice (figure 2C), but their treatment with both 200 and 400 µl extract led to an increase in sperm motility. Likewise, the mice treated with both extract doses exhibited higher sperm motility than those in the control group.

### Impact of the broccoli extract on Catsper1, Catsper2, and Arl4a gene expression with and without Cd-toxicity

**Figure 3:**
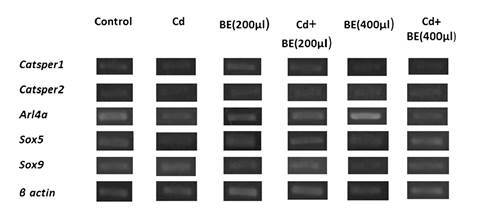
Relative gene expression determined by semi-quantitative RT-PCR. The expression of genes corresponds to the ratio of the target gene divided by the reference gene (β *actin*).

**Figure 4:**
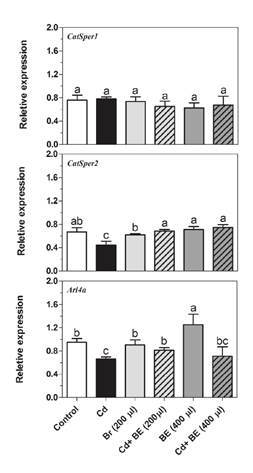
Transcript levels of *Catsper1* and *Catsper2* (genes involved in sperm motility) as well as *Arl4a* (gene involved in sperm count) in NMRI male mice. For experimental details, see legend in Figure 1. Relative gene expression was determined by RT-PCR compared to *β actin* as reference gene. Data represent the mean of three mice in each group (± SD). Different letters indicate significant differences among groups (p ≤ 0.05; Oneway ANOVA; Tukey´s HSD all-pairwise comparisons as a post-hoc test).

Figure 3 shows the relative gene expression by semi-quantitative RT-PCR while in figure 4 no significant difference in the *Catsper1* gene expression among groups is evident.

*Catsper2* gene expression in Cd-treated mice was significantly lower than in the other groups (Figure 4). However, there was no significant difference among extract-treated groups with and without Cd-toxicity and a closer look at the graph indicated that the *Catsper2* gene expression in the groups treated with 200 and 400 µl extract plus Cd was significantly higher than in the mice treated only with Cd (figure 4).

The *Arl4a* gene mRNA level was up-regulated (1.8 fold) in the group treated with 400 µl extract (figure 4) while its expression was clearly down-regulated in the group treated only with Cd but higher in the two groups treated with 200 and 400 µl extract plus Cd than in the mice treated only with Cd (figure 4).

### Impact of the broccoli extract on the gene expression of transcription factors SOX5 and SOX9 with and without Cd-toxicity

**Figure 5:**
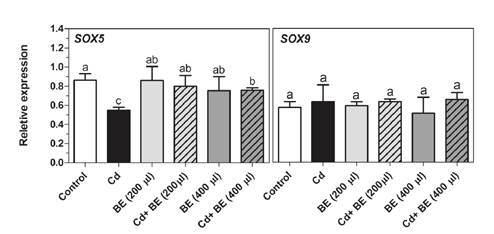
Transcript levels of *Sox5* and *Sox9* (transcription factors involved in sperm motility) in NMRI male mice. For experimental details, see legend in Figure 1. Relative gene expression was determined by RT-PCR compared to *β actin* as reference gene. Data represent the mean of three mice in each group (± SD). Different letters indicate significant differences among groups (p ≤ 0.05; Oneway ANOVA; Tukey´s HSD all-pairwise comparisons as a post-hoc test).

Figure 5 provides the experimental data on the gene expression of *Sox5* and *Sox9* transcription factors. Following the intraperitoneal administration of Cd, we detected a significant decrease in the *Sox5* gene expression. However, no significant differences were found in *Sox5* gene expression between extract- treated mice (200 and 400 µl) and the control group while it was significantly up-regulated in the extract plus Cd-treated mice compared with those treated only with Cd. Furthermore, *Sox9* gene expression appeared to be unaffected by Cd. Likewise, none of the extract-treated mice groups, both with and without Cd, showed significant differences in the *Sox9* mRNA level (figure 5).

## Discussion

We found few publications on the association between spermatogenesis and broccoli extract ^31^^,^^32^. In such context, we conducted the present study to determine the effect of this extract on the sperm factors and the expression of some genes whose relation to spermatogenesis has already been reported.

The most obvious and interesting finding (figure 2A) was the significant dose-dependent increase in sperm count among extract-treated groups even in the presence of Cd. Surprisingly enough the *Arl4a* gene expression also showed a similar increase in sperm count in extract-treated groups with and without Cd. ARL4 is a 22-kDa GTP-binding protein abundant in testes of pubertal and adult rodents. In mouse *Arl4*-null mutants (*Arl4* ^-/-^) the inactivation of the *Arl4* gene caused a significant reduction of testis weight and sperm count (30% and 60%, respectively) ^22^ and, in general, the association between testis weight and the *Arl4a* gene has been reported in the literature ^33^.

Genome-wide mapping in house mice revealed a significant association between SNPs located in the *Arl4a* gene and relative testis weight. However, no significant difference between body and testis weight among the six groups in our study was found. Similarly, Zhou, *et al.* did not find significant effects on body and testis weight in mice treated with broccoli seed extract (0.3, 1, and 3 g/kg body weight/day) for 30 days ^34^ and the broccoli seed extract LD_50_ in rats was >10 g/kg of body weight/day.

**Figure 6:**
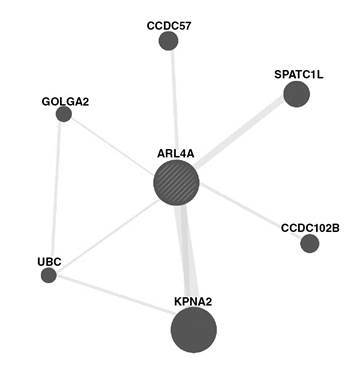
Protein-protein interaction between human ARL4A protein and six other proteins based on the GeneMANIA algorithm

Although the role of the *Arl4a* gene in mouse spermatogenesis was reported in 2002 ^22^, very little is currently known about its biological functions and spermatogenesis-related mechanisms. In this sense, we conducted an *in silico* analysis to find further evidence regarding its role in spermatogenesis and its binding proteins using the GeneMANIA algorithm. As shown in figure 6, *Arl4a* was bound to Spatc1l through the protein-protein interaction. Previously, it had been shown that Spatc1l maintains the integrity of the sperm head-tail junction and that Spatc1l knockout mice developed male sterility owing to the separation of sperm heads from tails ^35^. The *Kpna2* gene also presented protein-protein interaction with Arl4a and it has been reported that it is a key mediator of nucleocytoplasmic transport in the embryonic testis. *Kpna2* mRNA was identified in pachytene spermatocytes and round spermatids ^36^ and *Kpna2* amount markedly increased in both cells ^37^. Golga2, another protein physically binding to Arl4a, also plays a role in spermatogenesis. Han, *et al.* showed that the inactivation of *Golga2* caused male infertility in a mouse model. In *Golga2* ^−/−^ mice, acrosome and round sperm heads, characteristic features of human globozoospermia, were absent ^38^ and the cytoskeleton was disorganized in testes.

During spermatogenesis, the ubiquitin-proteasome pathway (UPP) plays a key role in facilitating the formation of condensed sperm and, therefore, deficient UPP blocks spermatogenesis. Ubiquitination occurs in different cell types during spermatogenesis, especially in spermatocytes differentiating into round and elongated spermatids and, even, in mature sperm in the epididymis ^39^. As shown in figure 4, ubiquitin C (UBC), one of the ubiquitination enzymes, can also physically bind to Arl4a.

Some studies have focused on the association between CCDC102B (coiled-coil domain containing protein 102b) and spermatogenesis. This protein is active in the assembly of the centrosome linker to maintain centrosome cohesion as it contributes to holding the duplicated centrosomes together and prevents centrosome separation. The regulation of the connection and/ or disconnection of two centrosomes is involved in several cellular processes, such as Golgi and cilia positioning ^40^. On the other hand, CCDC57 is another protein whose functionality has not been well-documented and it may play a role in the centrosome as it has a coiled-coil domain ^41^.

Interestingly, it was found that Arl4 has two separate promoters in the rat. Jacobs, *et al.,* showed that the mRNA transcription is under the control of the downstream promoter in most tissues while the upstream promoter seems to drive specifically the expression of Arl4 in adult testis ^42^. In fact, they recorded tissue-specific alternative splicing and promoter use in the *Arl4* gene. These findings provide insights into the molecular control of Arl4a protein in spermatogenesis. Apparently, it has several biological functions in different tissues and organelles, and its expression is regulated developmentally.

Broccoli accumulates selenium, consequently reducing the risk of several cancers ^43^^,^^44^; besides, it is required for spermatogenesis and male fertility ^45^. Selenium significantly increased the *Catsper1* and *Catsper 2* gene expression in adult male mice ^46^ and, therefore, it can enhance sperm mobility. The results of our study indicated that *Catsper2* was also up-regulated with the dose-dependent broccoli extract; however, no significant difference was detected in the *Catsper1* mRNA levels. We also observed an obvious increase in spermatogenesis using the extract, which is consistent with the selenium enrichment of broccoli and its positive impact on this process. Our finding is in line with those reported by Raeeszadeha, *et al*. who observed that the number of spermatogonia, primary spermatocytes, spermatids, and sperm significantly increased with the hydro-alcoholic extract of broccoli (300 mg/kg) in male mice after 42 days of treatment ^32^.

As reported in other studies, we found that sperm count, motility, and viability were reduced in Cd-treated mice while the *Catsper2* gene was down-regulated ^47^^,^^48^. However, our findings diverge from those reported by Mohammadi, *et al.* who observed a reduction of *Catsper1* mRNA level in Cd-treated mice ^48^. Of course, *Catsper1* gene expression is complex, its promoter is bidirectional and regulates the lncRNA (*Catsper1au*) expression. Indeed, *Catsper1au* is expressed in adult male mouse testis and can regulate gene expression during spermatogenesis ^49^. It may be that broccoli contains compounds that bind directly or indirectly to lncRNA regulatory proteins or binds to the *Catsper1au* target site or some other unknown phenomenon involved in *Catsper1* gene regulation.

Previous studies have established that both Sox5 and Sox9 transcription factors interact with the *Catsper1* promoter in HEK-293 cells ^50^. As shown in figure 5, the gene expression in *Sox9* was similar to that in the *Catsper1* gene, and no differences were observed among treatments in any of them, which agrees with Mata-Rocha, *et al.*’s findings. However, our findings indicated that *Sox5* gene expression was dramatically reduced in Cd-treated mice as compared with the control. A deeper look revealed that in the 200 and 400 µl extract-treated mice, *Sox5* mRNA level was slightly higher than in the Cd plus 200 and 400 µl extract-treated mice may be due to the other transcription factors or to the miRNA, e.g., miR-195, which targets Sox5 3’ UTR and impairs its expression ^51^.

Quercetin and kaempferol are the predominant flavonoids in commercial broccoli ^52^. Quercetin attenuates Cd-induced oxidative damage and apoptosis in granulosa cells from chicken ovarian follicles ^53^. In spite of the antioxidant properties of quercetin, a significant concentration-dependent conflicting effect on sperm viability and motility has already been observed ^54^^,^^55^, which is somewhat in line with our findings. Indeed, we observed that the broccoli diet affected positively the sperm count; however, it negatively impacted sperm viability, though increasing sperm motility slightly (figure 2). Raeeszadeh, *et al.,* determined that the total antioxidant IC_50_ of broccoli hydro-alcoholic extract is 278±14 µg/ml ^32^, a finding that probably explains the negative impact of broccoli extract on sperm viability and highlights the complexity of its antioxidant properties. Additionally, it is reported that the treatment with quercetin markedly inhibited nickel-induced global hypermethylation and DNA hypomethylation of the nuclear factor E2-related factor 2 (Nrf2) promoter ^56^, which may subsequently affect its downstream target genes. Kaempferol also inhibits DNA methylation by suppressing DNA methyltransferases ^57^.

It has been previously observed that kaempferol leads to the downregulation of DNMT3 (a kind of DNA methyltransferase) and modulates DNA methylation in cancer ^58^. Indeed, kaempferol alters 103 DNA methylation positions (hypo-methylation and hyper-methylation) associated with bladder cancer genes ^57^. Likewise, sulforaphane, another predominant ingredient in broccoli, increased *Nrf2* expression, a transcription factor, in prostate tumor cells through epigenetic regulation in mice ^59^. By the activation of Nrf2/ARE signaling pathways, sulforaphane prevented testicular damage in Cd-treated mice ^60^. Sulforaphane also reduced Cd-induced toxic effects on human mesenchymal stem cells and showed a significant recovery of cell viability ^61^. Despite supportive evidence on the impact of sulforaphane as an anticancer agent, the fact is that it also interferes with the T cell-mediated immune response ^62^ turning it into a double-edged sword.

There is a growing number of reports on the epigenetic regulation of broccoli ingredients and the stable and reversible mechanism regulating gene expression. Further research is needed to determine which compounds in broccoli extract stimulate spermatogenesis without a negative effect on sperm viability. Future studies on lower dosage would allow finding a suitable concentration of broccoli to avoid its negative effect on sperm viability and use it as a medicinal plant for male infertility.

In future studies, experiments using real-time PCR would offer a more precise view of gene expression alterations. Probably, *Homo sapiens*-based protein-protein interaction instead of *Mus musculus* do not reflect exactly what happens in mice; however, this *in silico* analysis definitely would be informative as it supports the role of Arl4a in the spermatogenesis process. Our results are promising for the pharmaceutical industry in its efforts to produce medications for infertile men.
